# Osteoclast Fusion and Fission

**DOI:** 10.1007/s00223-012-9621-6

**Published:** 2012-06-30

**Authors:** Ineke D. C. Jansen, Jenny A. F. Vermeer, Veerle Bloemen, Jan Stap, Vincent Everts

**Affiliations:** 1 Department of Oral Cell Biology, Research Institute MOVE, Academic Centre for Dentistry Amsterdam, University of Amsterdam and VU University, Gustav Mahlerlaan 3004, 1081 LA Amsterdam, The Netherlands; 2 Department of Periodontology, Research Institute MOVE, Academic Centre for Dentistry Amsterdam, University of Amsterdam and VU University, Gustav Mahlerlaan 3004, 1081 LA Amsterdam, The Netherlands; 3 Van Leeuwenhoek Centre for Advanced Microscopy, Department of Cell Biology and Histology, Academic Medical Centre (AMC), University of Amsterdam, Meibergdreef 15, 1005 AZ Amsterdam, The Netherlands

## Reply to Pavlos and Ng

To the Editor:

We have read the letter by Pavlos and Ng with great interest and thank the authors for their enthusiastic comments and support of our findings. We agree with Pavlos and Ng that the phenomenon of osteoclast fission and the possible participation of a relatively small mononuclear cell, as also noted by these authors, are very intriguing and need more attention in order to understand their role in the fission process and the underlying mechanisms. Pavlos and Ng note that in our work the process of fission took about 68 h, whereas they found that a similar process of fission occurred in only 1–2 h. Although we did monitor the cells by live cell microscopy for 68 h, in our experiments fission occurred within a few hours or even less, a result closely similar to the findings reported by Pavlos and Ng in their letter.

